# Processing, Characterization and Disintegration Properties of Biopolymers Based on Mater-Bi^®^ and Ellagic Acid/Chitosan Coating

**DOI:** 10.3390/polym15061548

**Published:** 2023-03-21

**Authors:** Carolina Villegas, Sara Martínez, Alejandra Torres, Adrián Rojas, Rocío Araya, Abel Guarda, María José Galotto

**Affiliations:** Center for Packaging Innovation (LABEN), Center for the Development of Nanoscience and Nanotechnology (CEDENNA), Technology Faculty, University of Santiago de Chile (USACH), Santiago 9170201, Chile

**Keywords:** Mater-Bi^®^, ellagic acid, chitosan, coating

## Abstract

Among the most promising synthetic biopolymers to replace conventional plastics in numerous applications is MaterBi^®^ (MB), a commercial biodegradable polymer based on modified starch and synthetic polymers. Actually, MB has important commercial applications as it shows interesting mechanical properties, thermal stability, processability and biodegradability. On the other hand, research has also focused on the incorporation of natural, efficient and low-cost active compounds into various materials with the aim of incorporating antimicrobial and/or antioxidant capacities into matrix polymers to extend the shelf life of foods. Among these is ellagic acid (EA), a polyphenolic compound abundant in some fruits, nuts and seeds, but also in agroforestry and industrial residues, which seems to be a promising biomolecule with interesting biological activities, including antioxidant activity, antibacterial activity and UV-barrier properties. The objective of this research is to develop a film based on commercial biopolymer Mater-Bi^®^ (MB) EF51L, incorporating active coating from chitosan with a natural active compound (EA) at two concentrations (2.5 and 5 wt.%). The formulations obtained complete characterization and were carried out in order to evaluate whether the incorporation of the coating significantly affects thermal, mechanical, structural, water-vapor barrier and disintegration properties. From the results, FTIR analysis yielded identification, through characteristic peaks, that the type of MB used is constituted by three polymers, namely PLA, TPS and PBAT. With respect to the mechanical properties, the values of tensile modulus and tensile strength of the MB-CHI film were between 15 and 23% lower than the values obtained for the MB film. The addition of 2.5 wt.% EA to the CHI layer did not generate changes in the mechanical properties of the system, whereas a 5 wt.% increase in ellagic acid improved the mechanical properties of the CHI film through the addition of natural phenolic compounds at high concentrations. Finally, the disintegration process was mainly affected by the PBAT biopolymer, causing the material to not disintegrate within the times indicated by ISO 20200.

## 1. Introduction

Nowadays, the main concerns in the food industry are oriented towards two different directions. The first one is related to food safety, preservation and quality assurance, and while second is the reduction in plastic packaging waste. One way to address these problems is the development of compostable and/or biodegradable materials to reduce environmental contamination without losing safe food [1]. Due to this, the development of biopolymers provides a pathway to accomplishing a sustainable environment by reducing dependency on non-renewable fossil fuel raw materials [2].

Currently, the most important polymers on the market are divided into three subgroups as follows: polymers based on renewable resources (starch and cellulose); biodegradable polymers based on biodegradable monomers (vegetable oils and lactic acid); and biopolymers synthesized by microorganisms [3]. Common degradable materials contain polyester-based biomaterials synthesized from natural raw polyethers such as polyhydroxyalkanoate (PHA) [4], including poly(3-hydroxybutyrate) and polylactide (PLA) poly-β-hydroxybutyric acid (PHB) [5], TPS [6] and commercial biopolymers, such as Mater-Bi^®^ (MB) [7].

These biopolymers are becoming more and more relevant in several investigations. Recently, phenolic extracts have been incorporated into PLA films to extend the shelf life of different foods [8]. Furthermore, ZnO nanoparticles have been developed as active functional nanocomposite films by compounding ZnO powder into TPS films [9]. On the other hand, modified cassava starch and tea polyphenols were compounded with PBAT to develop biodegradable active films [10]. In this context, within these biopolymers are some of a commercial character, such as Mater-Bi^®^, and biodegradable plastic produced by Novamont S.p.A. (Novara, Italy) developed in the 90s is currently used for different packaging [11]. Mater-Bi^®^ is a family of biodegradable and/or compostable polymeric formulations containing thermoplastic starches, which have good thermal stability and processability, and present high stretchability and toughness [12,13]. The morphological, mechanical, thermal, chemical and optical properties of this polymer have been studied [14], and they have also been used for the formulation of emulsions containing dissolved biopolymers both in the oil and water phases for in vitro hydrophilic and hydrophobic dual-drug release [15]. The anaerobic biodegradability of the commercial biopolymer has also been studied [16].

Moreover, another tendency is to focus on reducing the use of additives in foods, and growing interest in the consumption of minimally processed foods has motivated the development of new strategies for food protection. In this context, active food packaging systems have arisen as a new concept for food protection through the incorporation of active compounds (antimicrobial, antioxidant or antifungal) in a matrix polymer in order to increase its shelf life without affecting its quality and sensorial properties. Active compounds are derived from natural sources, such as plants, animals, bacteria, algae and fungi. These compounds have wide chemical diversity and different functions, which have allowed the study of their characteristics with the aim of exploring its applications in different sectors, such as agriculture and biomedicine [17].

Among the diversity of active compounds, there are phytochemicals that are mainly divided into two groups as follows: (1) phenolic acids that are characterized by having a ring in their structure; and (2) flavonoids that contain various hydroxyl groups associated with phenolic groups [18]. Ellagic acid (EA) is a polyphenol dilactone whose chemical structure is made up two lactone rings and two benzene rings, including two carbonyl groups and four phenolic hydroxyl groups [19]. As a polyphenol, ellagic acid is widely found in various soft fruits, nuts and other plant tissues [20]. As far as we know, ellagic acid has various biologically active functions, such as antioxidant functions, anticancer and antimutagenic properties, and inhibitory effects on human immunodeficiency virus [21]. Various investigations have focused on the study of the effects of the incorporation of compounds into different polymeric matrices in response to various requirements. For this case, ellagic acid has been used in homogeneous, translucent and flexible starch films for protection of UV rays and antibacterial activity against foodborne pathogens, such as Gram-positive and Gram-negative bacteria [22]. Additionally, the release of EA from zein films has been studied for use as wound dressings, evaluating its antioxidant and antimicrobial capacity [23].

As mentioned above, AE has advantageous properties and could be used for the development of active packaging [24]. In order to obtain active packaging with potential antimicrobial and/or antioxidant activity, it is necessary to establish a method for incorporating the compound into the polymeric matrix. Among these methods are processes such as extrusion, coating or coating, solvent dissolution or casting, electrospinning, supercritical impregnation, among others. In recent years, the technology of coating or coating has been widely used to generate materials with new and innovative properties. This technological and industrial process provides solutions to various sectors, ranging from packaging, consumer goods, medical products, and textiles to multinationals in the areas of transport and agricultural [25].

In the coating technique, an active agent is immobilized on the surface of the polymer matrix. The main advantage of this is that active agents can cover the inner layer of the package without interfering with the thermal or mechanical process, which results in the preservation of their highest activity in contact with the food surface [26,27]. Biopolymer-based coatings deposited on a plastic substrate represent an efficient technique and a sustainable approach for the development of innovative packaging and a reasonable alternative solution to plastic waste disposal issues [28]. Among the compounds used as coating phase agents is chitosan (CHI), which is a naturally occurring basic polysaccharide that is gaining consideration due to its innate antimicrobial activity, environmentally friendly nature, abundance and good film-forming ability among the most common biopolymers [29]. Its use is justified by its stable thermal and mechanical properties [30]. Furthermore, among various biopolymers, chitosan is a beneficial carrier for the controlled delivery of active ingredients, owing to its proven nontoxicity, absorption capability, biodegradability and biocompatibility [31].

Recently, chitosan has been used as an antibacterial material in packaging because of its certain antibacterial properties [32]. Nowadays, it has been used to develop nanosensors, showing excellent analytical behavior toward glucose sensing [33] as it also presents advantageous biological characteristic properties that make it an excellent biomaterial to be employed in skin tissue engineering [34]

In view of the above, the objective of this study is to develop a film based on a commercial polymer, incorporating a coating of chitosan with a natural active compound at two concentrations (2.5 and 5 wt.%). The formulations that obtained complete characterization were carried out in order to evaluate whether the incorporation of the coating significantly affects thermal, mechanical, structural, water-vapor barrier and disintegration properties.

## 2. Materials and Methods

### 2.1. Materials and Reagents

In this study, Mater-Bi^®^ (MB) EF51L from Novamont (Novara, Italy), ellagic acid (EA) powder from tree bark (C_14_H_6_O_8_, ≥95.0%) from Aldrich® Chemistry (St. Louis, MO, USA), sodium hydroxide (NaOH pellets, ≥99.0%) from Merk (Darmstadt, Germany), acetic acid (CH_3_COOH, ≥99.0%, Merk), and chitosan (CHI) at a low molecular weight with a degree of deacetylation (DDA) of 98% (Sigma-Aldrich, St. Louis, MO, USA) at a concentration at 2% solution in 1% of CH_3_COOH were used.

### 2.2. Preparation of Biopolymers (MB)

Films were obtained from Mater-Bi^®^ EF51L pellets via the extrusion process using a Scientific LabTech LTE20 twin-screw extruder (Samutprakarn, Thailand). The temperature profile was between 120 and 180 °C, with screw and feed speeds of 30 and 20 rpm, respectively.

### 2.3. Chitosan (CHI) and Ellagic Acid (EA) Active Coating Composition

**Figure 1:**
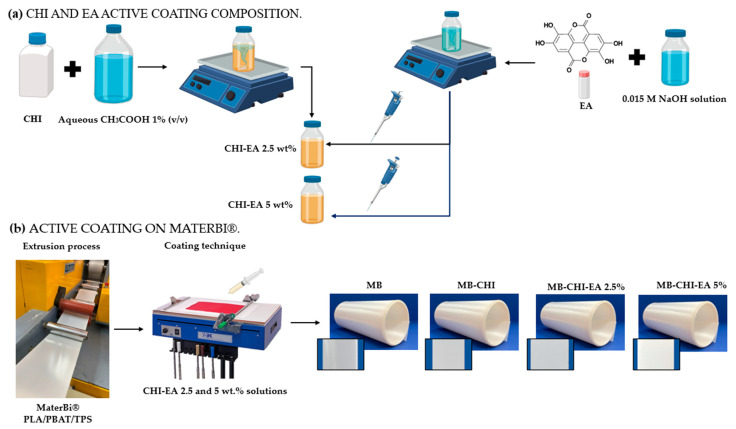
Schematic diagram of the process of obtaining active commercial biopolymers (**a**) coating composition and (**b**) incorporation active coating.

In Figure 1, a schematic diagram of the process of obtaining the active coating can be observed. Firstly, the coating was prepared by dissolving 2% (*w*/*v*) low-molecular-weight chitosan in aqueous acetic acid 1% (*v*/*v*). Simultaneously, EA solutions were prepared by dissolving EA in 0.015 M NaOH [22]. The mixtures of CHI and EA (2.5 and 5 wt.%) solutions were homogenized by mechanical stirring for 3 h at 25 °C. Before incorporating the active coating, corona treatment (Corona FT-800 equipment) was carried out with an intensity of 0.5 m volts at a speed of 1.8 m/s into MB. Finally, coating was performed using RK Print K303 equipment (Royston, UK).

### 2.4. Characterization of Biopolymers (MB) and Active Coating Biopolymers

#### 2.4.1. Attenuated Total-Reflectance Fourier-Transform Infrared (ATR-FTIR) Spectroscopy

FTIR spectra of the films were obtained using a Bruker Alpha spectrometer, equipped with a total-reflection diamond-crystal accessory (Bruker^®^, Platinum) with a resolution of 4 cm^−1^ over a wavenumber range of 4000 to 400 cm^−1^ with 24 scans. Spectra analysis was performed using OPUS^®^ Software version 7 (Billerica, MA, USA).

#### 2.4.2. Thermal Properties

##### Differential Calorimetry Scanning (DSC)

The assays were carried out by means of differential scanning calorimetry (DSC), using a Mettler Toledo DSC model 822e (Schwarzenbach, Switzerland) in a 0 to 300 °C temperature range. The heating scan of the samples was conducted at 10 °C min^−1^ and samples of 8–10 mg were placed in hermetically sealed capsules.

##### Thermogravimetric Analysis (TGA)

Thermogravimetric analysis (TGA) tests were performed with a Mettler Toledo Gas Controller GC20 Stare System TGA/DCS (Schwarzenbach, Switzerland). Samples of approximately 8–10 mg were heated from 25 to 500 °C at 10 °C min^−1^ under a nitrogen atmosphere.

#### 2.4.3. Mechanical Properties

Tensile strength, elongation at break and modulus of elasticity for each material were measured at room temperature with a Zwick Roell model BDO-FT 0.5 TH Tensile Tester, according to ASTM D-882 over 100% elongation. Tests were performed in rectangular probes (10 cm × 2.5 cm) at 25 °C and 50% relative humidity for 48 h before the test. Analyses were carried out with a 1 kN load cell. Initial grip separation was 5 cm and the crosshead speed used was 50 mm min^−1^. The results are the average of 10 specimens for each film.

#### 2.4.4. Water-Vapor Permeability

The water-vapor transmission rate was determined using permeability cells and films with an area of 4 cm^2^. Measurements were performed under ASTM F1249-90 at 37.8 °C and 97.3% relative humidity, recording the mass of the films daily using a Shimadzy Aux120 digital balance (Kyoto, Japan).

#### 2.4.5. Disintegration under Composting Conditions

Disintegration under composting conditions was performed by following the ISO-20200 standard [35]. Solid synthetic waste was prepared by mixing 10% of compost commercial, 30% rabbit food, 10% starch, 5% sugar, 1% urea, 4% corn oil and 40% sawdust, and it was mixed with water in a 45:55 ratio. Water was added periodically to the reaction container to maintain relative humidity in the compost [36]. MB, MB-CHI, MB-CHI-EA 2.5% and MB-CHI-EA 5% samples were prepared (square 2.5 × 2.5 cm), and they were buried 6 cm deep in plastic reactors containing solid synthetic wet waste. Each sample was contained in a textile mesh to allow their easy removal after treatment, while also allowing the access of microorganisms and moisture [37]. Reactors were introduced in an air circulation oven (Universal Oven UN110 Memmert, Schwabach, Germany) at 58 °C for 90 days. Aerobic conditions were guaranteed by periodical gentle mixing of the solid synthetic wet waste [36]. Extraction of the disintegrated samples was carried out from the day after the start of the process (day 0) during defined time intervals (1, 3, 7, 14, 21, 28, 36, 50, 57, 64 and 90 days), washed with distilled water, dried in an oven at 37 °C for 24 h and weighed. Photographs with digital camera (Canon IXUS 195. Amstelveen, The Netherlands) were taken to all samples once extracts from the composting medium for visual comparison. Meanwhile, the disintegration degree was calculated by normalizing the sample weight each day of incubation to the initial weight. In order to determine the time at which 50% of each film was degraded, disintegrability degree values were fitted using the Boltzmann equation [37].

### 2.5. Statistical Analysis

A randomized experimental design was considered for the experiments. Data analyses were carried out using Statgraphics Centurion XVIII (StatPoint Technologies, Warrenton, VA, USA). This software was used to implement variance analysis and Fisher’s LSD test. Differences were considered significant at *p* < 0.05.

## 3. Results and Discussion

### 3.1. Attenuated Total-Reflectance Fourier-Transform Infrared (ATR-FTIR) Spectroscopy

**Figure 2:**
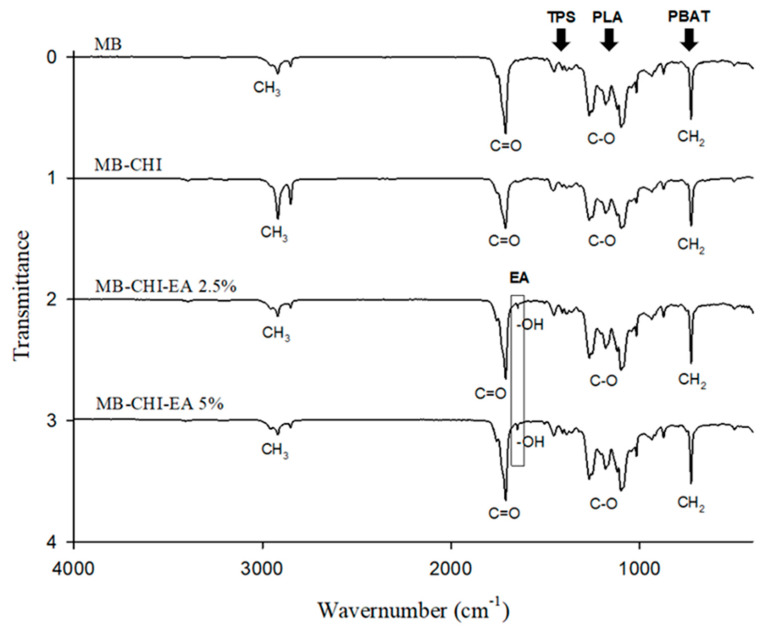
IR spectra of the biopolymers and active coating biopolymers.

Figure 2 shows that the MB FTIR spectrum reveals the presence of bands attributable to three polymers, namely poly (lactic acid) (PLA), poly-butylene-adipate-co-terephthalate (PBAT) and starch. The most characteristic peaks of PLA are at 2944 cm^−1^ and 2995 cm^−1^, attributed to symmetrical and asymmetrical vibrations of axial CH groups in saturated hydrocarbons (CH_3_) at 1082 and 1128 cm^−1^, where peaks of the C–O stretching bond of the polymer can be seen [38,39]. On the other hand, the characteristic functional groups of PBAT are located at 3000 cm^−1^ due to C–H stretching of the aliphatic and aromatic fractions, at 1720 cm^−1^ for the presence of C=O carbonyl groups in ester linkages and at 1274 cm^−1^, representing C–O in ester linkages. The bands recognized at 1574 cm^−1^, 1496 cm^−1^, 1456 cm^−1^ and 1019 cm^−1^ can be assigned to the stretching of phenylene groups, whereas the sharp band located at 720 cm^−1^ is characteristic of four or more adjacent –CH2– methylene groups. Furthermore, bending modes of benzene substitutes can be recognized in the spectral range of 700–900 cm^−1^ [14]. Finally, absorption bands related to the starch-based fraction can be found at 1445–1325 and 1250–900 cm^−1^ [38,40]. In addition, it is possible to observe a slight intensity increase in –NH and –OH stretching vibrations of chitosan when loaded with EA, indicating enhanced hydrogen bonding interactions involving –OH groups of EA with biopolymer –NH and –OH groups [41].

In addition, it is possible observe an increase associated with the bands at 3004–2830 cm^−1^ between the MB and MB-CHI biopolymers. This behavior could be due to the overlapping of νCH vibrations in the CH_3_, CH_2_ and =CH_2_ groups coming from biopolymers and chitosan coating [1] that incorporated active coating which causes the same band to decrease due to low affinity of EA in the hydrophilic coating.

The EA spectrum shows three bands at 3562 cm^−1^, 3468 cm^−1^ and 3149 cm^−1^, corresponding to the axial stretch of OH. The bands at 1656 cm^−1^, 1600 cm^−1^, 1710 cm^−1^ and 1157 cm^−1^ are associated with the aromatic C=C stretch, C–C stretch, C=O stretch and C–O–C, respectively. The band at 1348 cm^−1^ corresponds to the hydrogen bonds (HO–H). Finally, the band at 758 cm^−1^ indicates the presence of the phenyl ring [42].

### 3.2. Differential Calorimetry Scanning (DSC)

**Figure 3:**
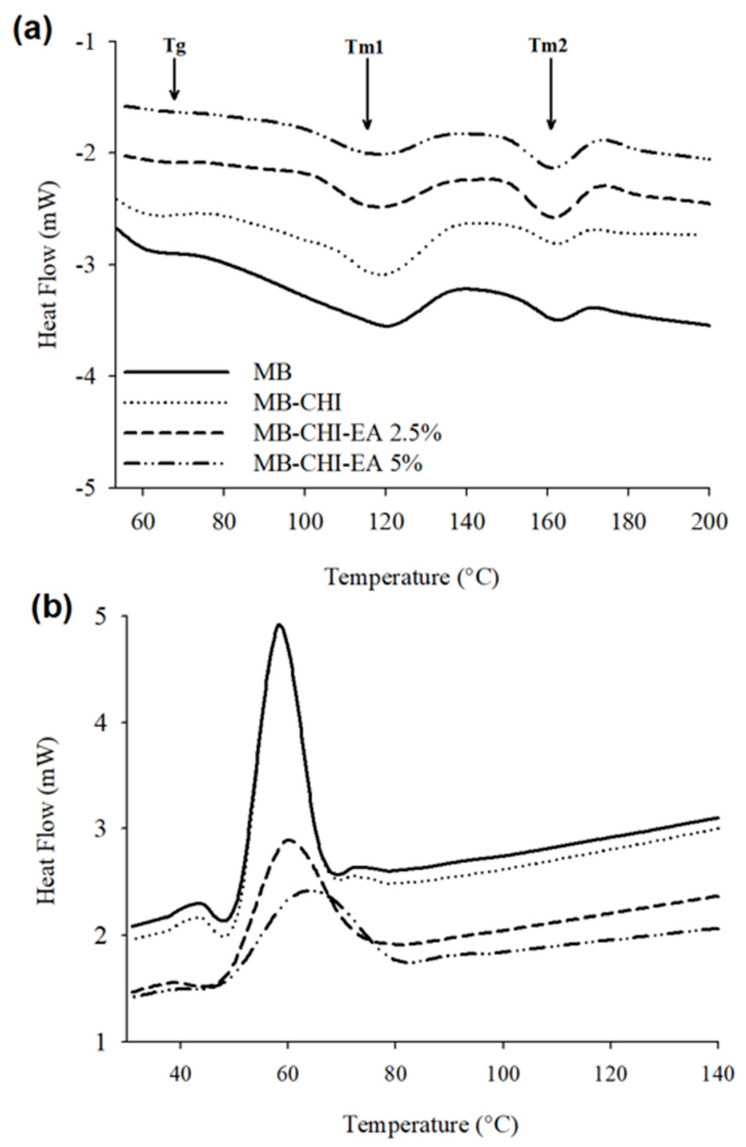
DSC thermograms during the (**a**) first heating scan and (**b**) cooling scan for all samples.

DSC analysis was performed to determine the effect of active coating on the thermal transitions of MB; the results are shown in Figure 3, and in Figure 3a, it is possible recognize three endothermic peaks. The first peak at ~61 °C is related the glass transition (Tg) of PLA [43], the second endothermic peak at ~119 °C corresponds to the melting of PBAT (Tm_1_), which is found between 115 and 125 °C [44], and the last peak ~160 °C (Tm_2_) is related to the melting of PLA [39]. With respect to the TPS, Surendren and coworkers indicated that the endothermic peaks between 165 °C and 180 °C could belong to the melting temperature (Tm) of thermoplastic starch (TPS) [45], while other research indicates that TPS has a wide melting range between 160 and 380 °C, which depends on the botanical origin and proportion of the starch the amylose/amylopectin ratio of the starch structure, in addition to the plasticizer used and the plasticizing conditions [46]. The behavior of Tg, Tm_1_, Tm_2_ and Tc is similar to that reported by Bianchi and Morreale (2023). They studied the thermal properties of PLA/PBAT, where it was possible to observe the first- and second-order temperatures associated with each polymer. With respect to PLA, two temperatures were identified, namely the glass transition temperature (60 °C) and the melting temperature (155 °C); meanwhile, the crystallization (78 °C) and melting (117 °C) temperatures correspond to PBAT [47]. These results are in accordance with the structural properties.

The incorporation of CHI-EA active coating caused a slight decrease in Tg, Tm_1_ and Tm_2_ due to the plasticizing effect. On the other hand, in Figure 3b, it is possible to observe a decrease in the crystallization peak, which could be due to the hydrogen bonding of the carbonyl group of the biopolymers and the OH and NH_2_ groups in chitosan; these interactions occur in the amorphous regions, suppressing the extent of crystallization [22].

### 3.3. Thermogravimetric Analysis (TGA)

**Figure 4:**
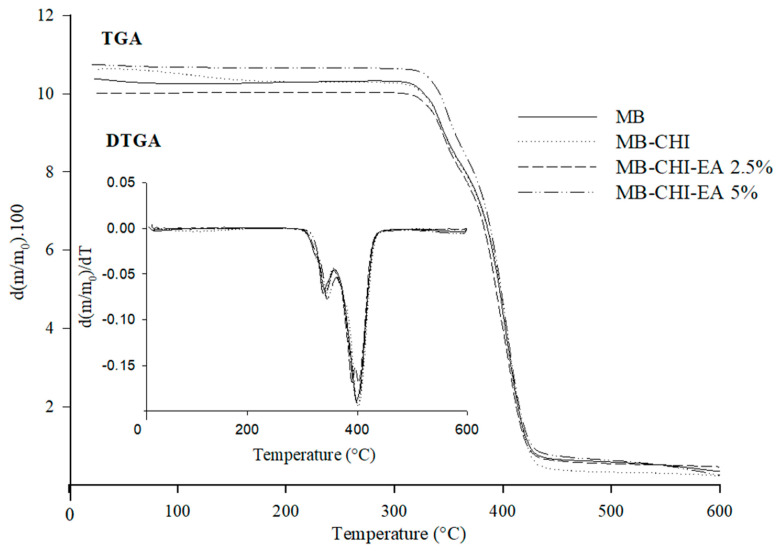
TGA and DTGA curves obtained for biopolymers and active coating biopolymers.

TGA is a very important technique used to evaluate the thermal stability of commercial biopolymers and active coating biopolymers. TGA and the first derivative (DTG) thermograms of different samples are illustrated in Figure 4. When evaluating the weight loss of MB, three mass losses are observed around 324, 336 and 400 °C, which describe the degradation of different biopolymers of the commercial sample.

Different researchers have been able to establish degradation temperature ranges between 50 and 320 °C for thermoplastic starch (TPS), which can be correlated with the elimination of water, glycerol and other low-molecular-weight compounds [48,49,50]. The temperatures range from 30 to 200 °C, which represents the evaporation of water adsorbed by starch and the plasticizers used, together with the evaporation of low-molecular-weight compounds [51] at ~320 °C, which represents the degradation of amylose and amylopectin [46].

It has been reported that the thermal degradation temperature of PLA ranges from 304 to 380°C [43,51]. Meanwhile, PBAT has a degradation temperature range between 390 and 410 °C [52,53]. Based on the literature, the first degradation inflection (324 °C) is attributed to TPS degradation, while the second (336 °C) corresponds to PLA decomposition and the third inflection (400 °C) corresponds to PBAT.

On the other hand, if the films with and without coating are compared, the latter show early minor weight loss (~100 °C), which is attributed to the desorption of moisture as hydrogen-bound water and acetic acid are present in the coating [54]. Furthermore, in films with coating, another inflection was observed close to 300 °C. Similar behavior was described by Vilela and coworkers (2017), who identified that chitosan presented a weight loss temperature at about 290 °C associated with the degradation of the CHI skeleton [22]. Meanwhile, Bonilla and coworkers associated this inflection to the degradation of the polymer structure, including the dehydration of saccharide rings and the decomposition of acetylated and deacetylated units at 298.3 °C [55]. The results point to the fact that the thermal stability of MB is not affected for CHI/EA active coating because of the presence of EA, due to most probably due to low loadings.

### 3.4. Mechanical Properties and Water-Vapor Permeability

The effect of the addition of a chitosan (CHI) coating layer, with different concentrations (2.5 and 5%) of ellagic acid (EA), on the mechanical properties of films based on Mater-Bi^®^ (MB) as a polymeric substrate was analyzed following the method detailed in Section 2.4.3.

Table 1 shows the tensile modulus (TM), tensile strength (TS) and elongation at break (EB) of the MB film, the MB biopolymer coated with chitosan (MB-CHI) and the MB biopolymer coated with CHI layers loaded with EA at 2.5% (MB-CHI-EA 2.5%) and 5% (MB-CHI-EA 5%). The MB monolayer film presented TM, TS and EB values consistent with the literature data [14]. TM and TS values for the MB-CHI film, composed of an MB substrate layer coated with a low-molecular-weight (MW) chitosan layer, were 15 and 23% lower than the values obtained for the MB film, respectively.

The effect of a CHI coating layer added over a polymer substrate using a solvent-based process on the mechanical properties of the resulting bilayer film depends on the balance between the plasticizing effect of the organic solvent on the polymer substrate and the reinforcing effect of the added CHI layer. Particularly, the polymer substrate reinforcement degree, achieved by adding a CHI layer is influenced by, among other factors, the molecular weight (MW) of CHI. Zhang and coworkers (2022) reported that the mechanical properties of CHI films, including TM and TS, significantly increased as the MW of CHI increased from 30 to 200 kDa [56]. In this context, effective increases in the mechanical properties of different biopolymeric substrates have been reported using CHI layers with medium and high MW, instead of using CHI layers with low MW. On the other hand, Fiore and coworkers (2021) reported a significant increase in TM and TS of poly acid(lactic) (PLA) films coated with a layer of CHI of medium MW [57]. In other work, such as that reported by Tanpichai and coworkers (2022), an increase in TM and TS of cellulose-based paper due to its coating with a layer of CHI of high MW was reported. The authors attributed this enhancement to the filling of voids between cellulose fibers with CHI and to the formation of additional hydrogen bonds between the anionic charges of cellulose and the cationic charges of CHI [58].

In this context, the decrease in TM and TS in the MB-CHI film, with respect to the monolayer MB film, could be related to the plasticizing effect of the CHI solution on the polymer structure and to the decrease in polymer substrate crystallinity. Both phenomena were reported in Section 3.2 and seem to be dominant over the reinforcing effect of adding a layer of CHI of low MW. On the other hand, it seems that CHI coating limits the extension capacity of the resulting MB-CHI film, decreasing EB from 670% to 568%. This behavior has been previously reported for other biopolymeric systems using a CHI layer as coating [57].

On the other hand, the addition of EA at 2.5% to CHI coating did not generate changes in the mechanical properties of the bilayer system (MB-CHI-EA 2.5%), with respect to the system without EA (MB-CHI). This fact agrees with the results reported by Vilela (2017), who reported production by solvent casting CHI films with EA up to 5% [22]. The opposite behavior was found in our study by adding EA at 5 % to the CHI layer. Particularly, TM and TS values for the MB-CHI-EA 5.0% film were higher than the values obtained for the MB-CHI system. The improvement in the mechanical properties of CHI films via the addition of naturally occurring phenolic compounds at high concentrations has previously been reported. Siripatrawan and Harte reported an increase in tensile modulus from 23.66 to 27.55 by adding green tea extract (GTE) up to 20%. The authors attributed this fact to the interaction between the CH matrix and phenolic compounds of GTE. Finally, the increase in EB in the bilayer system due to the addition of EA at 5% could be related to the plasticizing effect on the polymer structure [59].

Table 2 shows the water-vapor permeability for different developed samples. From these results, it can be observed that the water-vapor permeability of the MB film remained unalterable after the addition of CHI and the activation of CHI-EA coating. These results agree with the results reported for other biopolymers coated with CHI, such as cellulose-based paper [58], as well as with the results reported for biopolymers coated with a CHI layer loaded with naturally occurring phenolic compounds, such as PLA film coated with CHI, incorporated with rosemary essential oil at 2% [57].

### 3.5. Disintegration under Composting Conditions

#### 3.5.1. Visual Appearance of Films during Composting

**Figure 5:**
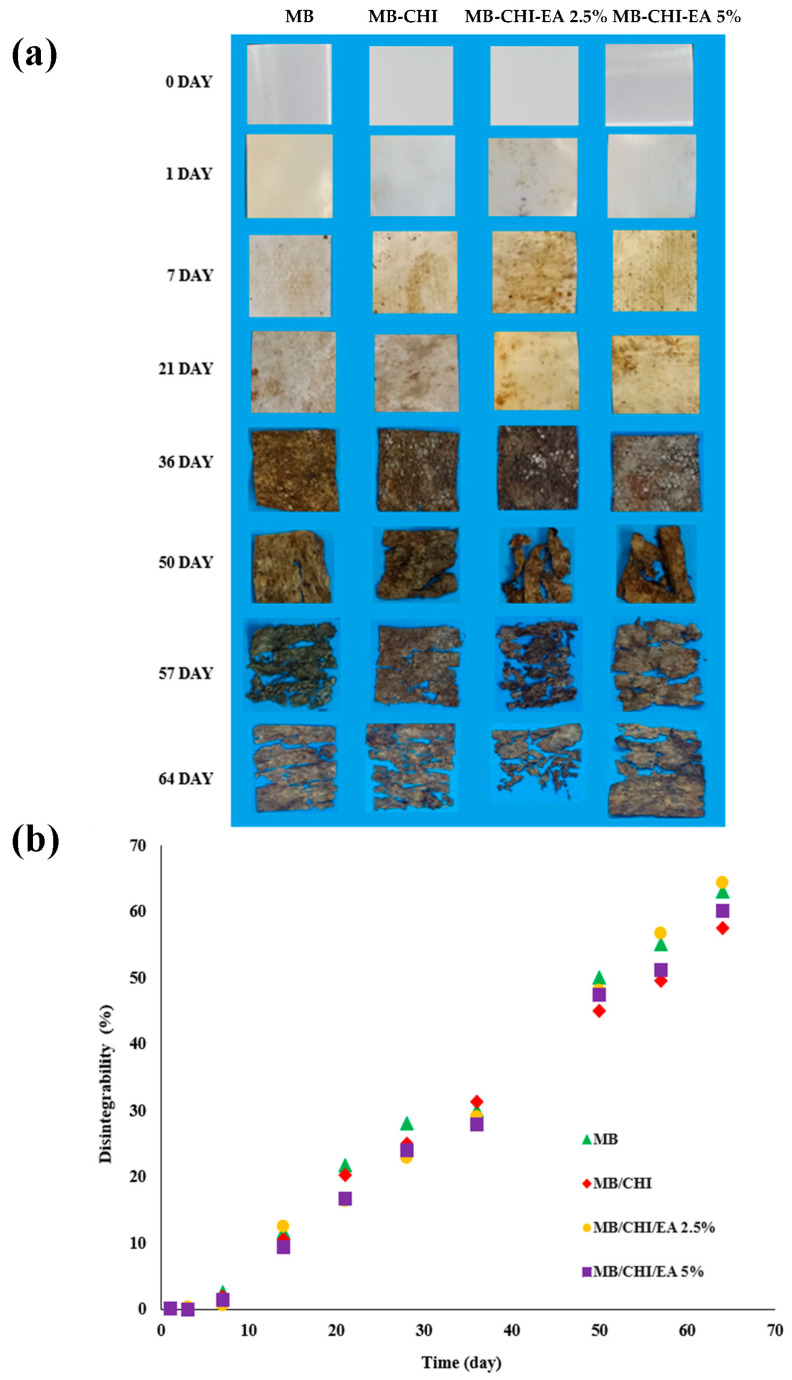
(**a**) Visual appearance of developed materials at different times under composting conditions; (**b**) film disintegration degree under composting conditions as a time function.

The visual inspection carried out of MB, MB-CHI and MB-CHI-EA (2.5 and 5%) after times of disintegration under composting conditions are shown in Figure 5a. From the results, it is possible to observe the slow rate of disintegration of the samples under composting conditions. After only 1 day of incubation, most of the formulations changed their color and became opaquer, losing transparency, which provoked a change in the refraction index of the materials as a result of water absorption and/or the presence of low-molecular-weight compounds formed by an enzymatic attack on the glycosidic bonds of the starch component of the Mater-Bi matrix [37].

After just 7 days, the samples presented signs of erosion, and aspect was greatly changed due to contact with the organic waste matrix presenting organic matter deposition on the surface. At day 50, some samples just started to break. The samples with the incorporation of CHI and CHI-EA (2.5 and 5%) that were still recoverable at 90 days presented slow degradation compared to other biopolymers.

#### 3.5.2. Disintegration Degree of Films during Composting

Visual observations were confirmed by calculating the disintegration degree in terms of mass loss as a function of incubation time using the Boltzmann function to correlate the sigmoidal behavior of the mass loss during disintegrability in the composting process, which is also presented in Figure 5b. From the image, it can be observed that all the samples reached a degree of disintegration of appropriately 70% with respect to their weight loss; however, these values do not comply with current legislation for biodegradable materials, which indicates that after a maximum time of 12 weeks, the samples must reach a degree of disintegration equal to or greater than 90% [35].

As discussed in Section 3.1, this commercial biopolymer is made up of PLA, TPS and PBAT. Regarding PLA, different research indicates that these biopolymers present a higher rate of disintegration when compared with the other biopolymers that make up MB. Villegas and coworkers (2019) studied the disintegration of PL. In their study, on day 7, small pieces of the analyzed polymers were collected due to physical and/or chemical degradation of the polymer, which occurred because it lost flexibility [46]. Due to this, PLA hydrolysis begins in the amorphous region of the polymer structure. Additionally, it has been reported that the intensity of the –C=O band increases with the composting time due to hydrolytic degradation, resulting in an increase in the number of carboxylic end groups in the polymer chains [36].

The degradation of TPS starts with non-enzymatic hydrolysis, which leads to a significant molar weight reduction, followed by enzyme action from the microorganisms present in the compost medium throughout the bulk of the polymeric matrix [60], as well as the biodegradation of starch-based polymers is a result of an enzymatic attack at the glucosidic linkages of the long-chain sugar units, leading to their breakdown into oligosaccharides, disaccharides and monosaccharides that are readily accessible to enzymatic attacks [61]. Sessini and coworkers (2019) studied thermal degradation and disintegrability under composting conditions of melt-processed blends based on ethylene-vinyl acetate and thermoplastic starch, such as EVA/TPS, as well as their nanocomposites, reinforced with natural bentonite. The results of the disintegration test showed that EVA/TPS blends and their nanocomposites presented positive interactions, which delay the disintegration of the TPS matrix in compost, thus improving TPS stability reaching 100% disintegrability in less than 60 days [60]. Meanwhile, blending biodegradable polymers, such as TPS, with non-biodegradable polymers, such as EVA, leads to an increase in compostable polymer percentage in partially degradable materials, giving a possible solution for the end-life of these materials after their use.

It is reported that the degradation of PBAT under composting conditions is considerably slower than that of PLA [62]. It is important to note that neat PLA almost degrades at 16 days, while a much longer incubation time is required to degrade PLA/PBAT blends and PBAT [63]. Recently, an investigation indicated that PBAT is a synthetic aromatic-aliphatic co-polyester with a molecular structure more complex than starch [64]. As such, it takes a longer period of time to be completely assimilated by microorganisms and transformed into stable products, and it is much more likely to subjected to process conditions, such as insufficient moistening content [65]. It has also been reported that PBAT disintegration is much slower and can take up to 12 weeks to disintegrate by 40% [66], although other authors have reported that PBAT takes 230 days to reach just 35% disintegration under the same composting conditions in this study (ISO 20200) because its structure must be hydrolyzed before microorganisms consume it as a source of nutrients [67]. On the other hand, Xie and coworkers (2023) developed large-size reed-reinforced PBAT composites with different filler degrees, and the properties and biodegradation behavior of the composites were investigated. With respect to the enzymatic degradation tests, it has been shown that the degradation rates of the composites were all greater than those of PBAT, and they all conformed to the surface erosion degradation mode. Furthermore, the main site of an enzymatic attack on PBAT during degradation is the ester bond, which breaks and increases the hydroxyl and carboxyl groups [68].

Similar results to this investigation were obtained by Aldas and coworkers (2021). They blended Mater-Bi^®^ NF866 with different additives to produce bio-based and compostable films for food packaging or agricultural mulch films. Disintegration was carried out in a test under composting conditions in which Mater-Bi^®^ reached 28% disintegrability over the 180 days of the composting test [16].

These results indicate that the disintegration of MB depends on the polymers that constitute it, but in this case, it stands out that TPS and PLA are easily biodegradable polymers, mainly due to the fact that starch-based materials are more available to microorganisms, improving the degree of disintegration; meanwhile, the high content of PBAT in the matrix explains the low disintegration degree of the material.

## 4. Conclusions

The effect of the addition of a chitosan (CHI) coating layer with different concentrations (2.5 and 5 wt.%) of ellagic acid (EA) on a commercial Mater-Bi^®^ (MB) biopolymer was studied. From the results regarding structural (FTIR) and thermal properties (DSC and TGA), it was possible to identify different temperatures and peaks associated with the three following biopolymers: PLA, TPS and PBAT.

With respect to the incorporation of CHI-EA, active coating caused a slight decrease in Tg, Tm_1_ and Tm_2_ due to the plasticizing effect; moreover, the addition of EA at 2.5 wt.% to the CHI layer did not generate changes in the mechanical properties of the system, while an increase in ellagic acid 5 wt.% improved the mechanical properties of CHI films by the addition of naturally occurring phenolic compounds at high concentrations. Regarding mechanical properties, the tensile modulus and tensile strength values of the MB-CHI film, composed of an MB substrate layer coated with a low-molecular-weight chitosan layer, were 15 and 23% lower, respectively, than the values obtained for the MB film. The effect of a CHI coating layer added over a polymer substrate via a solvent-based process on the mechanical properties of the resulting film depends on the balance between the plasticizing effect of the organic solvent on the polymer substrate and the reinforcing effect of the added CHI layer. Finally, during the disintegration process, all the samples reached a degree of disintegration of appropriately 70% with respect to their weight loss; however, these values do not comply with current legislation for biodegradable materials, which indicates that after a maximum time of 12 weeks, the samples must reach a degree of disintegration equal to or greater than 90%. PBAT is principally responsible for this slow degradation because it takes a longer period of time to be completely assimilated by microorganisms and transformed into stable products.

## Figures and Tables

**Table 1:** Mechanical properties for the different samples developed.

Samples	Thickness (µm)	Tensile Modulus—TM (MPa)	Tensile Strength—TS (MPa)	Elongation of Break—EB (%)
MB	109 ± 7 ^a^	286 ± 20 ^c^	31 ± 3 ^b^	670 ± 79 ^b^
MB-CHI	161 ± 5 ^b^	244 ± 15 ^a^	24 ± 2 ^a^	568 ± 54 ^a^
MB-CHI-EA 2.5%	171 ± 15 ^c^	335 ± 21 ^b^	25 ± 2 ^a^	565 ± 67 ^a^
MB-CHI-EA 5%	183 ± 20 ^c^	267 ± 22 ^d^	30 ± 3 ^b^	722 ± 94 ^b^

Mean value (*n* = 10) ± SD. Parameters in columns denoted with the same letters (^a–d^) do not differ statistically at the level of confidence (0.05).

**Table 2:** Water-vapor permeability for the different samples developed.

Samples	WVP (g/m^2^/día)
MB	7.4 × 10^−14^ ± 5 × 10^−15 a^
MB-CHI	4.9 × 10^−14^ ± 3 × 10^−15 b^
MB-CHI-EA 2.5%	8.20 × 10^−14^ ± 1, 8 × 10^−15 a^
MB-CHI-EA 5%	7.6 × 10^−14^ ± 7 × 10^−15 a^

Mean value (*n* = 10) ± SD. Parameters in columns denoted with the same letters (^a,b^) do not differ statistically at the level of confidence (0.05).

## Data Availability

Data sharing not applicable.
